# Words matter

**DOI:** 10.1038/s41390-022-02198-2

**Published:** 2022-07-19

**Authors:** Elena Fuentes-Afflick

**Affiliations:** University of California, San Francisco, CA USA

Diversity, equity, and inclusion have become defining issues for society and medicine. The domestic and global events that have occurred since 2020, particularly the murder of George Floyd,^1^ have galvanized the medical community to evaluate our role in the creation, perpetuation, identification, and eradication of health disparities and promotion of health equity. Researchers and scholars face the challenge of developing and implementing evidence-based approaches to defining comprehensive conceptual models, testing clear hypotheses, recruiting diverse study participants, and considering a range of methodologic issues in order to move our field forward in a constructive and deliberate fashion.

A recent commentary published in *JAMA Pediatrics* reported a major concern regarding the analysis and interpretation of race as a study variable.^2^ Drs Clark and French’s commentary, “Conflating race and genetics among newborns with neonatal abstinence syndrome,” highlighted two articles on neonatal opioid withdrawal syndrome (NOWS) and described the implications of the conflation of race and genetics. Based on an analysis of two papers, one published in the *Journal of Perinatology* and another published in *Pediatric Research*, Drs Clark and French highlighted an important methodologic issue in clinical research: the distinction between treatment that newborns received to treat NOWS and the treatment that newborns required to treat NOWS. In the absence of laboratory-based methods of diagnosing NOWS, clinicians rely on scoring systems, usually validated, to assess neonates’ symptoms and guide pharmacologic treatment. While scoring systems are intended to mitigate subjective assessments, the risk of inconsistent, even biased, assessment remains. As researchers, we must understand that our data may not be as objective as we assume and we must consider data validity within the overall framework of interpretation.

Drs Clark and French criticized the article in *Pediatric Research*^3^ for considering treatment received as an accurate measure of the treatment required and for attributing race-based differences in treatment for NOWS to genetics. According to Drs Clark and French, the manuscript “perpetuates racism by offering genetics as an explanation for racial treatment differences among newborns with NOWS”.^2^

The conflation of race and biology is common in the literature and significant because it can result in “misdiagnoses and underdiagnoses”.^2^ The risk of differential treatment is not restricted to neonates with NOWS; in a recent study of children with appendicitis, black children received less treatment for pain, which suggests, according to the authors, “a different threshold for treatment”.^4^ While standardization and validation testing of scoring tools will not single-handedly eliminate the risk of provider bias in clinical assessment, such efforts are important and must be a priority.^5^

The impact of genetic research has been amplified since the sequencing of the human genome.^6^ As highlighted by Drs Clark and French, authors frequently consider genetic factors in the context of racial or ethnic differences in health outcomes but a conceptual framework that attributes outcomes to genetics may perpetuate racism.^2^ For example, if structural or systems-level factors are associated with the outcome of interest but not included in the study, attributing causality to race or ethnicity would be incorrect and potentially harmful.^7^ Similarly, if racism is the cause or a contributor to health outcomes, attributing differences in outcomes to genetic factors, rather than racism, is conceptually wrong, harmful, and may divert attention from underlying social causes.^6,8^ There is an emerging focus on racism^2^ and we must continue to operationalize and incorporate racism into our studies.^8^

The fields of genomics and genetics have expanded our understanding of many important issues^6^ but the use of race in genetic research has been challenging and controversial for many years^8,9^ and some have described a legacy of scientific racism.^10^ For decades, investigators have grappled with the challenge of defining and evaluating population-based differences in health outcomes but there is no unifying conceptual framework.^6^ I support calls for the scientific community to create a mechanism to define evidence-based approaches to these complex and compelling issues, which have significant implications for science and policy.

In the context of the methodologic challenges associated with race and ethnicity, some have proposed analyzing ancestry as an alternative measure. Ancestry has been described as a “process-based concept,” whereas race is “a pattern-based concept”.^6^ The concept of ancestry places an individual into a broader context of geographic ancestry, rather than genetic ancestry,^8^ but there is no unified definition of the term and it may not fully address the shortcomings of race or ethnicity.^10^ Given the complexities of definition and interpretation, there is an urgent need for clarification and consensus on the topics of race, ethnicity, and ancestry to guide clinicians, researchers, and policymakers.

I support the statement, clearly articulated by Drs Clark and French,^2^ that race is a social construct. Based on this conceptual foundation, analytic frameworks must be updated to reflect the pathways and mechanisms that can be used to interpret racial- or ethnicity-based differences in health outcomes,^10^ including factors such as racism and social determinants of health. We must no longer permit casual or explicit attribution of genetic factors as explanations. Funders must carefully consider the analytic approaches they support and researchers must be guided by the most updated scientific models. Reviewers, editors, and publishers must critically consider every element, every word, of scholarly submissions and ensure that publications meet the highest standards of scholarship. Finally, the entire scientific community must achieve consensus on the scientific approach to analyzing variables such as racism, race, ethnicity, and ancestry.^6^

In the world of clinical care, research, scholarship, publication, and policy, words matter. The Editorial Board of *Pediatric Research* has been actively engaged in discussions about how to address health disparities and health equity. We recently created an editorial section to foster scholarship in this area, with a dedicated section editor, an approach that has been implemented by other journals.^11^ To address the important concerns raised by Drs Clark and French and to achieve our goal of eliminating health disparities and promoting health equity through research scholarship, we welcome your engagement.

## Data Availability

The references that form the basis of the commentary are listed in the references. There are no other data utilized in this manuscript.
